# Manual for transference work scale; a micro-analytical tool for therapy process analyses

**DOI:** 10.1186/s12888-014-0291-y

**Published:** 2014-11-18

**Authors:** Randi Ulberg, Svein Amlo, Per Høglend

**Affiliations:** Research Unit, Division of Mental Health, Vestfold Hospital Trust, PO Box 2169, 3125 Tønsberg, Norway; Department of Psychiatry, Vestre Viken Hospital Trust, Drammen, Norway; Division of Mental Health and Addiction, University of Oslo, Oslo, Norway

**Keywords:** Transference, Manual, Psychodynamic, In-session process

## Abstract

**Background:**

The present paper is a manual for the Transference Work Scale (TWS). The inter-rater agreement on the 26 TWS items was good to excellent and previously published. TWS is a therapy process rating scale focusing on Transference Work (TW) (i.e. analysis of the patient-therapist relationship). TW is considered a core active ingredient in dynamic psychotherapy. Adequate process scales are needed to identify and analyze in-session effects of therapist techniques in psychodynamic psychotherapy and empirically establish their links to outcome. TWS was constructed to identify and categorize relational (transference) interventions, and explore the in-session impact of analysis of the patient-therapist relationship (transference work). TWS has sub scales that rate timing, content, and valence of the transference interventions, as well as response from the patient.

**Methods:**

Descriptions and elaborations of the items in TWS are provided. Clinical examples of transference work from the First Experimental Study of Transference Interpretations (FEST) are included and followed by examples of how to rate transcripts from therapy sessions with TWS.

**Results:**

The present manual describes in detail the rating procedure when using Transference Work Scale. Ratings are illustrated with clinical examples from FEST.

**Conclusion:**

TWS might be a potentially useful tool to explore the interaction of timing, category, and valence of transference work in predicting in-session patient response as well as treatment outcome. TWS might prove especially suitable for intensive case studies combining quantitative and narrative data.

**Trial registry name:**

First Experimental Study of Transference-interpretations (FEST307/95). Registration number: ClinicalTrials.gov Identifier: NCT00423462. URL: http://clinicaltrials.gov/ct2/show/NCT00423462?term=FEST&rank=2.

**Electronic supplementary material:**

The online version of this article (doi:10.1186/s12888-014-0291-y) contains supplementary material, which is available to authorized users.

## Background

### Analyzing the process in therapy sessions

Analysis of the patient-therapist relationship (Transference Work; TW) is considered a core active technique in dynamic psychotherapy [1]. Transference intervention is a specific treatment technique which is believed to set in motion a chain of events assumed to bring about insight and dynamic change [2]. How to analyze and interpret transference patterns revealed during therapy and their impact on in-session process and long-term outcome, have been discussed. The historical development and recent empirical findings with regard to the concept of transference, definitions of transference interventions (TI) and the effects of transference work (TW) have recently been summarized [3,4]. While important initial contributions have been made, much remains to be studied empirically about how TI operates to influence the patient response (in-session) as well as the long-term outcome. The present paper is a manual for the Transference Work Scale (TWS), a psychotherapy process rating scale previously reported [5].

To identify and analyze in-session effects of therapist techniques in psychodynamic psychotherapy and empirically establish their links to outcome rely on adequate process scales [4-6]. Development of treatment manuals in psychodynamic psychotherapy and development of adherence rating systems have enhanced the research on psychotherapy outcome [6-8]. Using adherence rating systems, the research focus can be on specific techniques.

Adherence measures based on psychodynamic clinical theory can help identify TI, measure the immediate effects of TI and help analyzing the in-session process in psychodynamic psychotherapy. Different instruments for identification of therapist techniques have been developed and should be acknowledged. Some examples of process- and adherence scales are the Analytic Process Scales (APS) [9], Comparative Psychotherapy Process Scale (CPPS) [10], Psychotherapy Process Q-set (Q-set) [11], The Therapist Intervention Rating System (TIRS) [Piper, unpublished manual]. The Interpretive and Supportive Technique Scale (ISTS) [2], Psychodynamic Intervention Rating Scale (PIRS) [12], Patient Psychotherapy Process Scale (PPPS) [13], Comprehensive Psychotherapeutic Interventions Rating Scale (CIPRS) [7], Achievement of Therapeutic Objectives Scale (ATOS) [14], and Manual for process ratings [15]. The rating scales mentioned are useful, complex and detailed rating systems. Some of them have included items classifying TI. The aim for the researchers in the First Experimental Study of Transference Interpretations (FEST) [16,17] was, to develop a simple measure usable when limited recourses define the available time for transcription and rating. TWS is especially designed to identify the five TI categories defined in FEST [5]. The further goal when developing TWS was to construct a tool that would be helpful to explore facets of the timing, content, and valence of the therapist’s interventions as well as the response from the patient. TWS is previously presented [5]. The present paper is a description and guide for using TWS in process-ratings.

### First experimental study of transference interpretations (FEST)

The First Experimental Study of Transference-interpretations [16,17] aimed to measure the effects of TW in dynamic psychotherapy.

FEST was a randomized controlled trial where one hundred patients seeking psychotherapy for depression, anxiety, and personality disorders, were allocated to psychodynamic psychotherapy with low to moderate levels of TIs (the transference group; N= 52) or psychodynamic psychotherapy with no TIs (the comparison group; N= 48). The treatment was 45 minutes once a week, maximum 40 sessions. For the transference group, the specific techniques (i.e. categories of TI) were prescribed.

In FEST transference interventions are organized in five categories. The categories are not hierarchical. Category 1, 2, and 3 are interventions pointing at the transaction between the patient and the therapist and exploring the patient’s thoughts and feelings about the therapist and the therapy. These first three categories can be seen as preparatory interventions simply pointing at interaction between patient and therapist or encouraging the patient to explore thoughts, feelings and fantasies about the therapist and the therapy. Category 4 and 5 are interventions including connections between repetitive elements in the patient’s relationships with other persons out of therapy and the patient’s relationship with the therapist. These categories of transference work combine the relational (modernist) construction of transference (categories 1 through 3) with the traditional construct of transference in relationship (category 4 and 5). Interventions pointing at transference of genetic (historical) origins where early experiences and relationships with childhood caregivers are linked to the transaction between the patient and the therapist are included in category 5 [3,18]:

1. The therapist addressed transactions in the patient-therapist relationship (address transaction)
2. The therapist encouraged exploration of thoughts and feelings about the therapy and the therapist’s style and behavior (thoughts and feelings about therapy).
3. The therapist encouraged patients to discuss how they believed the therapist might feel or think about them (beliefs about therapist).
4. The therapist included him-/herself explicitly in interpretive linking of dynamic elements (conflicts), direct manifestations of transference, and allusions to the transference (linking therapist to dynamic).
5. The therapist interpreted repetitive interpersonal patterns (including genetic interpretations) and linked these patterns to transactions between the patient and the therapist (repetitive interpersonal pattern).

The outcome measures in FEST were the Psychodynamic Functioning Scales (PFS) [19], Inventory of Interpersonal Problems-Circumplex version (IIP-C) [20], Global Assessment of Functioning Scale (GAF) (Diagnostic and statistical manual of mental disorders, 1987) and Symptom Checklist- 90-R (SCL-90) [21].

The use of specific transference techniques differed significantly between the treatment groups using a 5-point Likert scale ranging from 0 (not at all) to 4 (very much). The average score was 1. 7 (SD= 0.7) in the transference group, and 0.1 (SD= 0.2) in the comparison group (t= 14.8, df= 58.2, p< 0.0005) [15,17,22].

In FEST no significant between-group differences were revealed. Both groups showed statistically significant change from pre-treatment to 3 year follow-up, with large effect sizes for all primary outcome variables. Contrary to expectation, moderator analyses showed that patients with a life-long pattern of poor relational functioning [16,17] profited more from therapy with TI than from therapy without. Patients with personality disorders (PD) profited more in therapy with TI than without TI [23]. The long-term effect of TW was mediated by an increase in the level of insight during treatment [24]. Women responded better to TI than men [25] and especially women with difficult relational functioning improved more with TI [26]. TI was most effective in the context of low alliance for patients with difficult relational functioning [27].

### Ethics

The Regional Ethics Committee for health region 1 in Norway approved the study protocol and the information given to the patients (FEST307/95). Written informed consent was obtained from each participant. Registration number: NCT00423462 URL: http://clinicaltrials.gov/ct2/show/NCT00423462?term=FEST&rank=2.

### Aims

The FEST research group has developed an in-session psychotherapy process rating scale focusing on TW; the Transference Work Scale (TWS). The items in TWS and results from interrater-reliability analyses are previously published [5]. The present manual aims to describe and elaborate the items in TWS, and describe in detail the rating procedure when using TWS. Clinical examples of transference work are included to guide the use of the scale.

## Methods

### Transference work scale

The Transference Work Scale (TWS) [5] was specifically developed to identify and explore in more detail the in-session-effects of TI. TWS is a therapy process rating scale constructed to identify, categorize and explore work with the transference. Development and inter-rater agreement has recently been reported and was good to excellent [5].

Subscales concerning identification, timing, categorization, content and valence of TIs as well as responses from the patient were included in TWS (Additional file 1). Greenson has emphasized the importance for the therapist to decide “…*what* he shall tell the patient, *when* he shall tell it, and *how* he shall do it.” Different authors have focused on different possible patient responses following the interpretation [5,28-30]. In TWS identification and categorization were based on the five categories of TI defined in FEST [16]. Items measuring timing assess the degree to which TIs connect naturally to the preceding clinical material and how precise and striking the TI is [29]. TWS has items describing the content of therapist interventions, such as dynamic conflict components (anxiety, defense, impulse/motives) and person components (parents, others) [31]. TW may trigger defenses [30,32]. This might be revealed when scoring items concerning the degree of therapist challenge or support, the patient’s attempts to avoid themes, level of emotional engagement, and whether the patient shows associations or self-reflections in the response [29].

TWS might be a potentially useful tool to explore the interaction of timing, category, and valence of transference work in predicting in-session patient response as well as treatment outcome and TWS seems suitable for intensive case studies combining quantitative and narrative data and also in combination with other process scales [5,33].

The clinical examples in the present manual are from FEST. Each example illustrates ratings on one or more of the 26 TWS-items. The examples are rated to illustrate specific scores on the different items. See also Table 1. However, when we could not find clinical examples in the available material, examples were developed for illustrative purposes (examples 13 through 17).

**Table 1:** **Transference work scale: overwiew of scoring examples**

**Item**	**Examples**				
**Identification**	All examples in Table 1 are transference interventions (TI)				
**Timing ITI** ^**a**^	**0**	**1**	**2**	**3**	**4**
6. ITI connect naturally	14,15,	13,17,	12, 36	8,16,	6,11,38
7. ITI precise/striking	15,16	13,14,17	12	8,36	6,11,38
**Category of the Transference Interventions (TI) in the Transference Work (TW)** ^**a**^					
8. Category 1	1,2 ,29,30,31,				
9. Category 2	3,4,11,19,22,23,32,38				
10. Category 3	5,6,20,21,28,33,34				
11. Category 4	7,8,18,24,35,36,37				
12. Category 5	9,10,12,25,26,27				
**Timing high category TI** ^**b**^	**0**	**1**	**2**	**3**	**4**
13. Connect naturally	14,15	13,17	12, 36	16	6,11,38
14. Precise/striking	15,16	13,14,17	12	36	6,11,38
**Content**					
15. Relation other T^c^	8,20,24,26	9,25,34	27,37	38	36
16. Relation other P^d^	9,27,26	20,34	8,24	25,37	36
17. Relation parent T^c^	8,20,34,36,37	9	8,24,25,27	6	26
18. Relation parent P^d^	9,20,24, 25,27,36,37,	34	26	6,21	23
19. Avoid themes	18,34	36	8,11,38	20,27,31	24,26,32
20. Symptoms T^c^	26	9,25,37	8,20,24,26, 27,34,36	4	16
21. Symptoms P^d^	8,37	9, 24,27,36,	25,26,34	12,20	7
**Valence**					
22. Supportive	22	9,20,27	18,19,23,24	34,36	6,21
23. Challenging	21,23	6,34	18,19,21,24,36,	22,27	9,20
**Response**					
24. Associations/self refl.	31	18,20,28	36	9,24,26	25,34
25. Cooperative	31	18,28	20	9,24,26,34,36	25
26. Emotional involvement	31	28	18,34	9,24,25,26,36,	20

*Note* ^*a*^Initial Transference Intervention (ITI). ^b^Timing of the first Transference Intervention with the highest category score. ^c^Therapist focusing on (T). ^d^Patient focusing on (P).

### How to rate with transference work scale?

#### Identification

The first aim when using TWS will be to identify the transference interventions and categorize them. Item 1 of the TWS is to decide whether there is any TI in the transcript or not. The items 2 through 4 are on identification of the first TI in the transcript and deciding where in the transcript this initial transference intervention is found. Item 5 is an item to identify the category of TI of the first occurring transference interventions (Initial Transference Interventions; ITI). The items 8, 9, 10, 11 and 12 are on whether the TW include TI of category 1, 2, 3, 4 or 5. See the examples 1 through 10 and more examples listed in Table 1.

#### Timing

Assessing timing with TWS includes items to decide to what degree do the ITI (Item 6) or the TI with highest category score (Item 13) connect naturally to the preceding clinical material, such as content, time context, allusions to the transference and other relevant issues. The other element of timing in TWS is how precise and striking the therapist’s ITI (Item 7) or the TI (Item 14) with highest category score is. See the examples 11 through 17 and more examples listed in Table 1.

#### Content

Characteristics of the content of the TW might influence the patient response (in-session) as well as the long-term outcome. Therefore items concerning content were included in TWS. The items are mainly organized in pairs on whether the patient or the therapist includes certain themes in their turns of talk in the transference work segment:

“To what degree does the therapist refer to the patient’s relation to others?” (Item 15).

“To what degree does the patient refer to the patient’s relation to others?” (Item 16).

With “others” we understand other people than the parents and the therapist (i.e. spouse, friends, colleagues).

“To what degree does the therapist refer to the patient’s relation to parental figures?” (Item17).

“To what degree does the patient refer to the patient’s relation to parental figures?” (Item 18).

Parental figures are parents or other adult people replacing parents during early childhood. Other people representing parental objects at present (i.e. teacher, boss) will not be rated as parental figures.

“To what degree does the therapist point out the patient’s attempt to avoid themes in the session in order to control unpleasant emotions and thoughts?” (Item 19).

Item 19 is the only question in TWS covering elements of defense.

“To what degree does the therapist refer to the patient’s symptoms?” (Item 20).

“To what degree does the patient refer to the patient’s symptoms?” (Item 21).

Symptoms are rated when psychological and somatic complaints including reduced functioning as well as problematic personality traits are mentioned. Discussing problems will not always be rated as symptoms. See the examples 24 through 27 and more examples listed in Table 1.

#### Valence

The valence subscale is aimed to help explore whether the therapist is challenging or supportive in the TW [29,30].

“To what degree does the therapist make use of supportive interventions?” (Item 22).

Supportive interventions include affirmation such as showing respect, acceptance and acknowledgement. The therapist’s contributions in the dialog are characterized by gratifying the patient, i.e. make the patient feel good rather than anxious in the session, or praise the patient. The therapist might provide information and guidance, engage in problem solving strategies, or offer explanations that locate the responsibility for the patient’s difficulties outside him/herself.

“To what degree is the therapist challenging in the interventions?” (Item 23).

Challenging interventions might be provocative interventions potentially awaking unpleasant emotions such as anxiety, shame and guilt in the patient. However, a challenging intervention does not need to be an unfriendly intervention and might also evoke a feeling of being met and recognized. See the examples 18 through 23 and more examples listed in Table 1.

#### Response

The patient response to the therapist’s TI is the in-session outcome. Scoring the response with TWS is aimed to measure the immediate effect of TI shown by the patient’s associations or self-reflections as well as the patient’s level of active cooperation/withdrawal and emotional aspects of the response.

“To what degree does the patient express associations and/or self reflections in the TW? “ (Item 24).

“To what degree does the patient show active cooperative engagement?” (Item 25).

“Identify with the patient: What is the highest level of emotional involvement?” (Item 26).

See the examples 24 through 26 and more examples listed in Table 1.

#### Step by step rating with TWS

1. One rating sheet is used for each segment/session when rating with TWS (Additional file 1). The items 1, 3 – 5, and 8 – 12 are rated with Yes or No. The other items are rated on a 5-point Likert scale ranging from 0 (not at all) to 4 (very much).
2. Choose the material to rate: To rate with TWS, transcripts from full sessions or segments of sessions are used. If possible, audio recorded sessions can be used in combination with the transcripts.
3. Read through the whole transcript.
4. Decide whether there is TI(s) in the transcript. If no TI is found, no further rating with TWS on the transcript can be performed (Item 1).
5. Identify and categorize the first TI (Initial Transference Intervention; ITI) in the transcript (Items 2–5). The segment to be rated is the segment in the transcript/session that starts with the first identified TI from the therapist and continues until the end of the transcript/session (i.e. transference work segment). The patient-therapist interaction preceding the ITI is not included in the rated segment.
6. After identifying the ITI the timing of this first TI in the transcript should be decided and rated (Items 6 and 7).
7. When more than one TI is identified, decide the category of each of them and rate the presence/not presence of each of the five categories in the transference work segment on the items 8–12. These five category items are rated with Yes or No. (Please see the examples 34 and 38).
8. One TI can build up and last for multiple therapist utterances (Please see the examples 9, 25, 27 and 34). Sometimes more TIs will be distinctly revealed, while sometimes successive therapist turns of talk constitute one TI.
9. Timing of the TI with the highest category score: If a TI with a higher category score than the category of the ITI is identified later in the transcript, rate it’s timing on items 13 and 14. If more than one TI with higher category score than the ITI is identified, choose the TI with the highest category score. If the ITI is followed by more than one TI with higher but similar categories, rate the timing of the first occurring high category TI in the segment. For example if ITI is a category 2 intervention and later in the transcript two more TIs of category 4 are identified, rate the timing of the first occurring category 4 TI. (Please also see example 26 and 36).
10. The content, valence and response are rated in the segment beginning with the ITI and continuing to the end of the transcript (transference work segment).

### Clinical examples of transference interventions rated with TWS

The following clinical examples are from the FEST-study. However, when using transcripts from transference therapies in FEST we could not find interventions sufficiently illustrating poor timing. Therefore the examples 13 through 17 are not from real therapies, but have been developed for illustrative purposes. The parts of the dialogues constituting the therapist’s transference interventions are italicized.

### Identification– clinical examples rated with TWS

Clinical examples of TIs with different categories are presented. More examples are listed in Table 1.

#### Example 1

This is an example of a TI of category 1 where the therapist sitting together with the patient is addressing transactions in the patient-therapist relationship (Item 8):

T *So here we are now.*

#### Example 2

This is also an example of a TI of category 1(Item 8; addressing transaction). The therapist points at the fact that the patient is telling something to the therapist:

T *Now you’re saying it to me and what you’re saying is perfectly clear.*

#### Example 3

Here is an example of a TI of category 2 (Item 9; thoughts and feelings about therapy). The therapist encourages the patient to explore what expectations the patient has towards the therapist:

T *You say you miss getting clearer advice and feedback from me? What do you feel about that?*

#### Example 4

This again is an example of a TI of category 2 (Item 9; thoughts and feelings about therapy). The therapist focus directly on the patient’s symptoms (score 3 on Item 20; symptoms) when encouraging the patient to explore feelings towards the therapy:

T *As we discussed, the therapy ends at the end of the month. But the symptoms and the troubles you sought help for, you still struggle with. What does it mean for you that you haven’t seen results yet?*

#### Example 5

Here is an example of a TI of category 3 (Item 10; beliefs about therapist):

T *It might be that you dread coming and talking because I might think that what you’re talking about isn’t an important subject, or that I am critical in some other way.*

#### Example 6

Another example of a TI of category 3 (Item 10; beliefs about therapist) where the therapist encourages the patient to discuss how the patient believes the therapist thinks about the patient:

P Towards other people I’ve had a polished façade; a strong barrier against being sad and helpless so people can see it. But here I have indeed shown this side of myself to you. There is not very much more to embellish here.

T *How do I see you?*

#### Example 7

The present patient-therapist interaction is an example of a TI of category 4 (Item 11; including therapist in dynamic) where the therapist in the last turn of talk, directly includes him/her in an interpretation of the patient’s internal dynamic and transference. The patient directly refers to own symptoms (score 4 on Item 21; patient refers to symptoms):

P When I sit with somebody, I get that bad feeling of it being my fault that it’s quiet. That she or he thinks I’m lame because I don’t have anything to say.

T Mm mm.

P That I take the blame for it being quiet.

T *Exactly. That’s interesting because that will be the situations where it’s the two of you and here we are the two of us talking together.*

#### Example 8

This shows an example of a TI of category 4 (Item 11; linking therapist to dynamic). The therapist to a low to moderate degree points at the patient’s attempt to avoid themes in the session. Item 19 (avoiding themes) is rated with 2 because the therapist is not very confronting, but more asking and encouraging the patient to deepen a theme:

P I notice that I’m very anxious about hurting my boss.

T *So you feel that you have to tread a bit carefully at work?*

P Mm

T *You told me that you were dreading the session today a bit and you also mentioned that your sleeping troubles had increased. Maybe you experienced it as hard to come and tell me that those problems actually had gotten worse, because it might be that I got hurt or disappointed?*

#### Example 9

Example 9 is a category 5 TI (Item 12; repetitive interpersonal patterns). The therapist in this segment points at a repetitive pattern in the patient’s emotions towards the father, important other people outside therapy as well as the therapist. Thus, through the therapist’s turn of talks, a category 5 intervention builds up. The therapist to a low degree refers to the patient’s relations to others and parents (score 1 on the items 15 and 17). Both therapist and patient to a low degree refer to the patient’s symptoms (score 1 on the items 20 and 21):

P I’m so stressed out today because I came late for this session.

T So what you’re saying is that you shouldn’t put me in such a squeeze.

P Maybe, but it has probably most to do with not living up to the ideal expectations.

T *Who’s expectations?*

P My own, ultimately.

T *What kind of expectations could I have?*

P That I show up on time, or else I can end up in discredit with you.

T *The way it was at home with your father if you didn’t accurately keep all agreements, and you have told that you feel the same way this year towards your boss.*

P Yes, I think others have expectations that I have to hurry up and fulfill. I do see that it’s my own expectations.

#### Example 10

Here is an example of a TI of category 5 (Item 12; repetitive interpersonal pattern). The therapist interprets a repetitive interpersonal pattern and links the patient’s tendency not to say “no” to mother and other people in the present life and links this to the transaction between the patient and the therapist:

P My mother called this morning. I immediately interrupted her and told her that if it wasn’t very important, I had no time talking now. I hung up and even though I had lots to do. I got a terribly bad conscience.

T *You told about your difficulties with saying “no” at work and protecting yourself in a better way. You couldn’t “hang up” neither when talking with colleagues, nor your mother or father because you were anxious about being rejected or punished. However, here you managed to tell me that our next session had to be changed because of your meetings at school and work.*

### Timing – clinical examples rated with TWS

In the following clinical examples of the timing of TIs are presented. More examples are listed in Table 1.

#### Example 11

The TI is of category 2 (Item 9; thoughts and feelings about therapy). Example 11 is an example of very good timing. The example is therefore scored with 4 on the items 6 and 13 (naturally connecting) and with 4 on the items 7 and 14 (precise and striking). The therapist to a low to moderate degree points at the patient’s attempt to avoid themes in the session. Item 19 (avoid themes) is scored with 2:

P I have always been proud of being independent, to be able to take care of myself. It has been a good feeling, but maybe I also have anxiety about being in debt to others, in a sense.

T *How is that towards me?*

P I haven’t thought of that (laughs).

#### Example 12

This example shows moderately good timing and is therefore scored with 2 on the item 6 and 13 (naturally connected) and with 2 on the items 7 and 14 (precise and striking). The patient focuses on symptoms (score 3 on Item 21). The TI is of category 5 (Item 12; repetitive interpersonal patterns). The TI links the repetitive interpersonal patterns to the transaction between the therapist and the patient:

P I’m trying to keep my spirits up by thinking about the times when I have managed to set limits for others, but I feel that it’s hopeless when I see what big problems I actually have with such setting of limits.

T Yes, you have given examples of such problems both at work and with your family.

P I have to tell them early on and I haven’t been able to do that.

T What do you want to tell me?

P Tell you? I don’t know. One time I said you were too close. I felt pressurized.

T *Yes, as you remember I heard your signal. You do say that your colleagues at work listen to what you are saying, too. But maybe you have to shout louder to your brother and be clearer with your boss if they are to hear you.*

#### Example 13

This is an example from the beginning of therapy. The TI is slightly connected to the preceding material (1 on the items 6 and 13; naturally connected). However, the intervention seems theoretical driven and is not successful in tuning in on the patients level of description of the situation and capacity for insight. Therefore the TI is rated as very little precise and striking (score 1 on the items 7 and 14):

P: Since starting in therapy here three weeks ago, I feel less anxious. Especially when you open the door and call for me, I can notice this.

T: *When you strongly emphasize feeling safe talking with me, could that mean that you feel insecure in relation to me? Unconsciously you are afraid of being rejected by me. You fear that you are not an interesting patient the same way you experienced your mother not being really interested in you, but only used you for her own needs.*

#### Example 14

This is an example showing poor timing and is also a highly disaffiliate comment; score 0 on the items 6 and 13 (naturally connecting) and score 1 on the items 7 and 14 (precise and striking):

P: I’m sorry. I have to cancel a session. It’s a session in the end of next month. I’m going to a meeting in Tokyo. I understand I should inform you of any cancellation as early as possible. I have previously always managed to change the time for meetings. I have tried to change the date for this meeting too. However, this time my boss told me that finding another time was “completely impossible”.

T: *Three months ago I cancelled two sessions and then came the Easter holiday which meant nearly one month with no treatment. It’s obvious that at least partly your cancellation is connected to this. You are unconsciously aggressive towards me because of this rejection. Therefore you now reject me as you experienced my cancellations as rejections. What do you think about that?*

#### Example 15

This is an example showing poor timing; score 0 on the items 6 and 13 (naturally connecting) and score 0 on the items 7 and 14 (precise and striking). The therapist’s intervention does not link to the preceding turn of talk from the patient:

P: Last week I told you about the possibility of a new job. Means a lot to me. As you know, my present job is only temporary. We are two applying for this job. After the session here, I’m going directly to an interview. I’m really afraid. I think I will make a fool of myself and suffer another defeat. Then my family has no income.

T: *A couple of months ago, you mentioned that when you were five years old, you broke your arm in a car crash. Your father drove the car. Unconsciously you blamed yourself for the accident because you were angry at your father. Now you direct this aggression towards me by inviting me to discuss your situation at work, your job interview, and your financial situation. What could I say? I can hardly have any opinion.*

#### Example 16

The present example shows a TI that connects naturally to the preceding clinical material (score 3 on the items 6 and 13), however, it is not at all precise and striking (score 0 on the items 7 and 14). The intervention takes into account what the patient just said, but is diffuse. The therapist focuses on the patient’s symptoms (score 4 on Item 20):

P: I felt insecure and sad about what we just talked about. I felt you were distanced, weren’t really interested and don’t actually care. I felt rejected.

T: *I wonder if you wish me to be more like a loving mother – to be caring whatever you want to discuss. I think it’s interesting that you don’t reflect on this and find it strange that you aren’t angry at me when you feel I reject you.*

#### Example 17

The timing of the TI in example 17 is poor. On TWS the TI is rated with 1 on the items 6 and 13 (naturally connecting) and 1 on the items 7 and 14 (precise and striking):

P: I‘m thinking of the fact that today is the last session. Thinking about how I will manage on my own makes me insecure.

T: *In your situation it must be connected with your challenges concerning assertiveness. You might also be relieved to be able to turn away from me.*

### Content– clinical examples rated with TWS

See ratings of the examples 4, 6, 7, 8, 9, 11, 12, 16, 18, 20, 24, 25, 26, 27, 31, 32, 36, and 38 also listed in Table 1.

### Valence – clinical examples rated with TWS

Clinical examples of the valence of the therapist’s interventions in TW are presented. More examples are listed in Table 1.

#### Example 18

The TI is of category 4 (Item11; including therapist in dynamic). By describing the patient’s feelings of not being interesting for other people, the therapist draws connections that might be challenging (score 2 on Item 23). The therapist does however, not point at the patient’s attempt to avoid themes in the session. Therefore Item 19 is scored with 0. Even though the TI is a little to moderately supportive (score 2 on Item 22), the patient to a low degree shows cooperative engagement (1 on Item25), and to a low extent expresses associations and/or self-reflection (1 on Item 24). However, he/she shows some emotional involvement (2 on Item 26):

T *You experience that I’m not interested in you and that others, for instance your teachers, aren’t interested either. Maybe you also feel that your boyfriend will lose his interest when he discovers who you really are. When that’s the way you think about yourself, it’s no surprise you are anxious and have a lump in your throat.*

P Yes, but how to relax. If I fail the exams now it will be a big defeat, disappointment, over and over again.

T You have to remember that your problems concentrating are caused by you not being well, but having a severe depression. You have experienced repeated severe episodes of it, ever since the teen years. It’s important to understand why it feels like this for you.

#### Example 19

The therapist’s TI is a little to moderately supportive (score 2 on Item 22) with elements of critical accusation and is therefore rated as challenging (score 2 on Item 23). The TI is a category 2 intervention (Item 9; thoughts and feelings about therapy):

T *You’re completely direct and clear in what you’re asking me about, but you bring it up only after I had kind of ensured you that it was all right if you said it. What do you think about that?*

#### Example 20

The TI is of category 3 (Item 10; beliefs about therapist). The therapist points at the patient’s attempt to avoid themes in the session and therefore Item 19 is scored with 3. The therapist TI is very little supportive (score 1 on Item 22) and very challenging (score 4 on Item 23). The patient shows a low degree of associations/self reflections in the response (1 on Item 24), is moderately cooperative (score 2 on Item 25), and shows a high degree of emotional involvement (score 4 on Item 26):

P During your summer holiday I can do just what I want. There will be no one who cares If I want to walk into the woods and take my life.

T *You wonder perhaps how much I care if you want to go into the woods and take your life?*

P If I just could get a telephone number to a human I could call if something happens.

T So you want a place to turn to? That I could give you a note with a telephone number.

P I would probably not use it, but felt that I at least have it.

T I should provide for you while I was away on holiday? That would have been good for you.

P It’s not about you. You are not a part of my private life.

T It is important for you when I’m away.

#### Example 21

This is a TI of category 3 (Item 10; beliefs about therapist). The therapist TI is very supportive (score 4 on Item 22) and only a little challenging (score 1 on Item 23). The patient focuses on the relationship with his/her mother (score 3 on Item 18). The example is scored with 4 on items 6 and 13 (Timing; connect naturally) and 4 on items 7 and 14 (Timing; precise and striking):

P I have such a bad conscience after my mother’s funeral because I had earlier wished that she was dead because it would have made life easier for me.

T *Of course you wished that she had been dead because it’s clear that it would have been considerably easier, in many ways. But you really can’t allow yourself to have such thoughts or feelings, even though they are highly understandable. When you have told me this today, what do you now think that I think of you?*

#### Example 22

The therapist’s TI is not at all supportive (score 0 on Item 22), however, quite challenging (score 3 on Item 23). The TI is of category 2 (Item 9; thoughts and feelings about therapy). The TI has, however, elements of category 3 where the therapist encourages the patient to discuss how he/she believes the therapist might feel or think about the patient. However, in the present example, the therapist asks if the patient wants advice and is not asking if the patient wonders what the therapists thinks about the patient:

P As I have told you, we had a discussion at home. I wonder what others would have done in that situation?

T *So then you wonder perhaps what I would have done or what I would have advised you to do?*

P Yes

T *Could you imagine what I could possibly do in that situation or advise you to do?*

P If I could imagine that?

T Yes, if you could imagine what that could be. Maybe you also have thoughts on why you want to know it or get my advice?

P It’s to confirm that I acted right.

T So when you feel you acted right, you still want a confirmation.

P Yes, I wonder what others would have done. Asked someone at work but she didn’t manage to put herself into my situation. I’m thinking that if I hear that what I do or think is right, then I feel safer regarding the future. Then I don’t become afraid of regretting later. Then I kind of know that I have done or thought the right thing.

#### Example 23

The therapist’s TI is a little to moderately supportive (score 2 on Item 22) and not at all challenging (score 0 on Item 23).The patient refers to the relationship with his/her father (score 4 on Item 18; relationship with parents). The TI is of category 2 (Item 9; thoughts and feelings about therapy):

P I wish I could stay away a couple of days more after the weekend.

T *Does our appointment disrupt you?*

P No, I was supposed to work.

T Yes, you called, so we moved our appointment from yesterday to today.

P That feeling is great. That I can speak up without feeling guilty. I was at my father’s place on Sunday. He is a farmer. I pointed out my views about livestock farming that he didn’t agree on, but I stuck with my opinion.

### Response – clinical examples rated with TWS

Clinical examples of the patient’s in-session response to TIs are presented. More examples are listed in Table 1.

#### Example 24

In the TI the therapist refers to the patient’s relations to his/her mother and the patient’s symptoms (score 2 on the items 17; relation to parents and 20; symptoms). The therapist is quite supportive but also challenging (score 2 on the items 22 and 23). The patient responds with referring to a moderate degree to others (score 2 on Item 16) and distinctively expressing associations and/or self-reflections (3 on Item 24), shows active cooperative engagement (3 on Item 25) and emotional involvement (3 on Item 26). The TI is of category 4 (Item11; including therapist in dynamic):

T *It’s like you’re rejecting having a personal opinion on how we can facilitate the therapy in practice.*

P Yes.

T *Just like you initially turned away from, that there was nothing more to get from yourmother?*

P Yes. I’m thinking about how damaged we are. I have had little contact with my sister, but she has used to be with me on Christmas Eve. One year I was mad at her and didn’t invite her, but last year she was there. She has been going to therapy for years. I called her recently and invited her for Christmas.

#### Example 25

Example 25 is a TI of category 5 (Item 12; repetitive interpersonal pattern) consisting of successive utterances from the therapist. In the TW the therapist to a low degree refers to the patient’s relations to others, parents and symptoms (score 1 on Item 15, 2 on Item 17, and 1 on Item 20). The patient refers to others (score 3 on Item 16) and symptoms (score 2 on Item 21), responds with distinctively expressing rich associations and/or self-reflections (4 on Item 24), and shows active cooperative engagement (4 on Item 25) and emotional involvement (3 on Item 26):

T *You present criticism on the design of the research program that the therapy is a part of. That is fine, but in that context you notice how hard it is for you to ask about something for your own sake. It’s much easier for you to be loyal to the research program, even if you think the design is bad.*

P Yes, it’s hard for me to ask about something for myself, I see that.

T *You have earlier given examples of that in relation to your father during your teens.*

P Yes, it’s probably the same now at work with my boss. For instance last week, but then I managed to be clearer about my needs with him. It was thought provoking what you said earlier about my relationship with the others at work. Yes, I could probably be an enthusiastic spokesperson for them towards the boss.

T *You could probably be a dangerous adversary both for your father and the boss.*

P Yes, it’s not a coincidence that I earlier in my career life for many years have been a trustee and the employees’ representative and led negotiations about wages on behalf of the employees.

#### Example 26

The therapist strongly points at the patient’s attempt to avoid themes in the session in order to control unpleasant emotions and thoughts (score 4 on Item 19) and focus on the patient’s relation to dad (score 4 on Item 17;relation to parents) but not on symptoms (score 0 on Item 20). The therapist’s turn of talks constitutes TIs of category 2 (thoughts and feelings about therapy) and 3 (beliefs about therapist). However, throughout the segment, a category 5 intervention (Item 12; repetitive interpersonal pattern) is built up. The first TI would be the initial TI (ITI) of category 2. The category 5 intervention will be the TI with the highest category score. The patient responds by expressing associations and/or self-reflections (3 on Item 24), shows active cooperative engagement (3 on Item 25) and emotional involvement (3 on Item 26).

P I have had a nice summer vacation.

T *You have been free from me, has that been a vacation?* (Category 2).

P It has been a blessing. No, I don’t know. I am always uneasy on my way here, maybe because it’s a process of confrontation.

T *Has today been like that as well?* (Category 2).

P Yes, I was actually in doubt about what I would bring up today, that the themes I was thinking about weren’t that essential, central. Maybe it’s an exaggerated expectation about what results this treatment will bring.

T *It could be that you’re dreading that I have exaggerated expectations?* (Category 3).

P No, absolutely not.

T *So, you dreading has nothing to do with me, but with coming here?* (Category 2).

P I think part of the problem is based on that discussion with dad. Because it evokes a feeling, but not as strong as if I had talked about a sexual problem.

T So your relationship with dad is also emotional even if it’s not as taboo as feelings about sexual life.

P Yes

T *Now the thought about dad is appearing here and it could be that since I also am a man and possibly the same age as your dad, this results in you transferring some of your experiences with dad to me. You’re possibly thinking that now I can be as critical as dad or in another way put you in a vulnerable situation or the likes, as you experienced him doing.* (Category 5)

P I haven’t thought about that. At least I can’t recognize that feeling.

#### Example 27

Successively the therapist’s intervention builds up to a TI category 5-intervention (Item12; repetitive interpersonal pattern). The therapists points at the repetitive pattern of not speaking up against his/her mother and boss as well as the therapist. The therapist is a little supportive and quite challenging in the TW (score 1 on Item 22 and 3 on Item 23). The therapist refers to the patient’s relations to others (score 2 on Item 15), parents (score 2 on Item 17), and symptoms (score 2 on Item 20). However, the patient does not refer to others or parents and only to a low degree to own symptoms (score 1 on Item 21). The therapist points at the patient’s attempt to avoid themes in the session (score 3 on Item 19).

P I have a loyalty I have to take into account at work. For the time being it’s not a problem because I have stayed outside of the conflict.

T *So the boss has claims on your complete loyalty also when it concerns events that don’t have anything to do with the job in particular. If the boss thinks something, you should think the same. And not speak up against him/her. The same way as with your mother when you previously were helping her in the shop. You said that you actually disagreed completely, but acted loyally.*

P Yes I’ve had a high respect for authority.

T *It could be that there are some things you have a hard time saying to your mother, or asking her about?*

P Yes, that’s clear.

T *And the same thing would apply to your boss?*

P Mm yes

T *And the same would apply here, I would think?*

P MM mm. Yes

T *And on what do you disagree – or what are you thinking that doesn’t get past your lips?*

P No.

T There you go!

P I would wish to have an opportunity to either have some follow-up sessions after the agreed final date or to be able to call you if something acute should happen. I would wish for that.

#### Example 28

The patient responds to a low degree with expressing associations and/or self-reflections (1 on Item 24), shows a low degree of active cooperative engagement (1 on Item 25) and little emotional involvement (1 on Item 26). First in this example, one sees a category 2 TI (thoughts and feelings about therapy) followed by a category 3 TI (Item 10; beliefs about therapist):

T *You have mentioned that the sessions here are important to you, something to hold on to and that it’s sad that the therapy is concluded in a month. But how do you think of the relationship that you and I have?* (Category 2)

P It’s a given time of the week in a given time period and it’s important for me during the process I’m in now.

T *You have said that you have begun to be hesitant about the conclusion of the therapy. You have mentioned that a thought has struck you that I think you’re a little tiresome, but not so much that it’s not endurable. Is that so?* (Category 3)

P Yes, I guess I have sort of thought about that.

### More clinical examples rated with TWS

#### Example 29

The TI is of category 1 (Item 8; address transaction):

T *You’re saying it very clearly here to me, are you just as clear towards him?*

#### Example 30

Here the TI is of category 1 (Item 8; address transaction):

T *You smile a little now when you’re saying it to me.*

#### Example 31

The therapist points directly at the patient’s attempt to avoid themes in the session (score 3 on Item 19). The patient responds with not expressing associations and/or self-reflections (0 on Item 24), nor showing active cooperative engagement (0 on Item 25) nor emotional involvement (0 on Item 26). The TI is of category 1 (Item 8; addressing transaction):

T *You talk about problems at work and at the same time you’re saying that you consider taking your own life. When you say that to me, it seems as a cry for help.*

P If it’s a cry for help, I do not know.

T You confuse me.

#### Example 32

The therapist to a high degree points at the patient’s attempt to avoid themes in the session. The Item 19 is scored with 4. The therapist encourages the patient to explore feelings about the therapy and the therapist. The TIs is of category 2 (Item 9; thoughts and feelings about therapy) and 3 (Item 10; beliefs about therapist):

T *What we talk about now, does it become just an unnecessary, foolish discussion, or can it help you?* (Category 2).

P No….. In a way it just becomes words. What you talk about, it doesn’t really help. I don’t see the connection there. I think it is silly to be so damned positive all the time. I don’t want to be positive if I don’t think it’s clearly justified.

T *You think I’m too positive?* (Category 2)

P I think so. To be perfectly honest.

T *So I’m fake?* (Category 2)

P You’re not fake, but…

T Manipulating?

P Yes, a little maybe. Like therapeutically manipulating.

T *I say things I don’t mean?* (Category 3)

P I think so.

T *How is it to have a therapist who is like that?* (Category 2)

#### Example 33

The TI is of category 3 (Item 10: beliefs about therapist). The therapist encourages the patient to discuss how he/she might feel or think about him/her:

P When someone asks me why I do it, then it’s like I have to find an explanation, an excuse for me doing it.

T *How is it when I say:“How can that be then?” Do you feel you have to have an explanation or excuse that I can accept?*

P Yes, no. An explanation, not necessarily an excuse.

T You owe me an explanation because you experience that it is important for you that I accept you?

#### Example 34

In this example the TI is of category 3 (Item 10; beliefs about therapist). Rating the content in the TW showed that the therapist and the patient to a low degree refer to the patient’s relation to others (score 1 on the Items 15 and 16). The patient mentions relation to parents (score 1 on Item 18). They both to a moderate degree refer to the patient’s symptoms (score 2 on the Items 20 and 21). The valence of the therapist’s contribution to the TW is supportive interventions (score 3 on Item 22) combined with a low degree of being challenging (score 1 on Item 23). The patient to a high degree expresses associations and/or self reflections in the response and shows cooperative engagement (score 4 on Item 24; score 3 on Item 25). The patient’s emotional involvement is moderate (score 2 on Item 26).

T *When I now ask you a question about what you said, might it be that you feel that I don’t accept you?*

P Yes, so then I have to find a good explanation.

T When I ask you such questions as now, I will sometimes be curious, but also have a wish to understand you. But first and foremost I am interested in what you think about it, so that we together can look at it closer. For example concerning the fact that you are constantly troubled with a bad conscience.

P Yes, I don’t understand why I go around excusing myself for everything, that I don’t take enough care of my mother and my brother, because I do take more care of my mother than most people.

T Than everyone.

P No, I don’t know about that, but if I don’t do it I make an excuse about not having the time for instance.

T Your sense of responsibility is so great that it fills every room in your life. Only when you have a valid reason you can make it smaller.

P It’s interesting that you say that because at work today I experienced the feeling that “It’s going to blow soon”, maybe I get fired. But when I then checked out something with my boss I got an almost disturbingly positive feedback. It was a very positive experience – meaningful.

T Maybe it helped you to trust that not everyone needs an excuse from you?

#### Example 35

Example 35 is an example of a category 4 TI (Item 11; including therapist in dynamic).

T *So you’re actually telling me something else than you’re telling him? Because to him you don’t tell that you miss the contact with him. What would happen if you were as open towards him as you now are towards me?*

P Exactly. So I should at least also be able to tell him that I miss the contact between us. That is probably not such a bad idea.

#### Example 36

Here the therapist’s interventions are building up with elements of a category 4 TI (Item 11; including therapist in dynamic). The timing of the TI is good (score 2 on items 6 and 13; naturally connecting and score 3 on items 7 and 14; precise and striking). The content in the TW is on the patients relation to others (score 4 on Item 15 and 4 on Item 16). The therapist and the patient refer to the patient’s symptoms (score 2 on Item 20 and 1 on Item 21) and the therapist points to a little degree on the patient’s attempt to avoid themes (score 1 on Item 19). The patient responds with associations/and or self-reflections, shows cooperative engagement and emotional involvement (Score 2 on Item 24, and 3 on the items 25 and 26):

T Then we start up again first on the Wednesday in the New Year.

P Yes, January the third

T *Yes. How does it feel that there won’t be any therapy session during the Christmas week?*

P I am not happy about that. This has become a fixed point that I look forward to, a sanctuary. For Christmas I travel home where the whole family is gathered, with all our family dramas and very direct confrontations.

T *Will you feel all alone with it?*

P Yes

T *You have nobody you can trust to speak with there?*

P I have a close friend there, but this is too far from her reality and she can’t catch it. So there are things I don’t tell, because then she might have gone around thinking about it.

T *Would that have been so bad? It is a friendship that has lasted a while, a friendship where one can carry a bit of the worries for each other.*

P Maybe I exaggerate, but I think like that.

T *We have talked earlier about a central point for you, that you are left standing very alone, can’t share it with others.*

#### Example 37

This is an example of a TI category 4 (Item 11; including therapist in dynamic). The therapist and the patient refer to the patient’s relations to others (Score 2 on Item 15 and 3 on Item 16). The therapist to a low degree refers to the patient’s symptoms (score 1 on Item 20):

P I wonder what others would have done in my situation, but I don’t get the answers in these sessions.

T By me you mean?

P Yes

T What do I think you should do?

P My wife had a problem and she said that she would certainly ask the others in the group about it.

T *Yes, your wife attends group therapy with 6–7 others who she can ask and get advice from. Here you are referred to one person, namely me, who doesn’t want to give you any advice. What do you feel about the fact that you think so much about what others would do and if others feel that what you do is OK?*

#### Example 38

The timing of the TI is very good (4 on the items 6 and 7). The TI is of category 2 (Item 9; thoughts and feelings about therapy) and category 4 (Item 11: including therapist in dynamic). The category 2-intervention would be the initial transference intervention (ITI), while the category 4-intervention would be the intervention with the highest category score. The transference work segment to be rated with TWS would begin from the first (category 2)-intervention. The therapist to a moderate degree points at the patient’s attempt to avoid themes in the session. The Item 19 is therefore scored with 2. The therapist focus on the patient’s relation to other (score 3 on Item 15):

P It’s been 3 weeks since I was here, I haven’t missed it, but it feels very good to come here today.

T *You say it feels good to come. Maybe that is something you want to talk about. You could also have dreaded coming because then you have to talk about difficult things*.(category 2)

P Oscar and I have decided to move apart from each other. I’m quitting my job in 3 months and moving back, home to Germany.

T *So you’re leaving the job, Oscar and me. How does that feel for you?* (category 4).

## Results and discussion

The Transference Work Scale was specifically developed to identify and explore transference work. The five categories of TI defined in FEST were used in TWS to delineate and operationalize the construct. How to use TWS was illustrated with clinical examples for each of the 26 TWS-item.

TWS is promising with regard to achievement of inter-rater agreement. Raters of TWS do not necessarily need to be experts, and there might be limited need for extensive training to achieve good inter-rater reliability [5]. Since TWS is a focused and short process measure, the scale can probably be used for creating datasets from a larger number of sessions or segments of sessions. TWS might prove especially suitable for intensive case studies combining quantitative and narrative data, and might also be used in combination with other process rating tools such as Structural Analyses of Social Behavior [5,33,34]. Ratings with Transference Work Scale might contribute to shed additional light on the associations between TI and in-session and long-term outcome in psychodynamic psychotherapy.

## Conclusion

The Transference Work Scale was developed based on distinct definitions of transference interventions and transference work. TWS might be a useful tool to explore the interaction of timing, category, and valence of transference work in predicting in-session patient response as well as treatment outcome. TWS might prove especially suitable for intensive case studies combining quantitative and narrative data. The manual includes rich clinical material to illustrate ratings on each item on the TWS.

## Additional file

Additional file 1:
**Transference Work Scale (TWS).**

## Abbreviations

ATOS: Achievement of therapeutic objectives scale
APS: Analytic process scales
CIPRS: Comprehensive psychotherapeutic interventions rating scale
DSM-IV: Diagnostic and statistical manual of mental disorders, fourth edition
FEST: First experimental study of transference–interpretations
CPPS: Comparative psychotherapy process scale
FWC-24: Feeling word checklist–24
GAF: Global assessment of functioning
GSI: Global severity index (total mean score of symptom checklist–90)
IIP-C: Inventory of interpersonal problems–circumplex version
ISTS: Interpretive and supportive technique scale
PFS: Psychodynamic functioning scale
Q-set: Psychotherapy process Q-set
PIRS: Psychodynamic intervention rating scale
QOR: Quality of object relation score
TIRS: Therapist intervention rating system
TI: Transference intervention
TW: Transference work
TWS: Transference work scale
