# Supplementary material for: Manual for transference work scale; a micro-analytical tool for therapy process analyses
Source: BMC Psychiatry. 2014 Nov 18;14:291. doi: 10.1186/s12888-014-0291-y (PMC4236796; doi:10.1186/s12888-014-0291-y)
Supplement: Additional file 1: — Transference Work Scale (TWS). [file 12888_2014_291_MOESM1_ESM.doc]

**Additional file 1**

**Transference Work Scale (TWS)[5]**

**Identification**

1. Are there any transference interventions in the transcript from

patient________,session________and segment________? YES NO

*If YES, answer the following questions:*

2. What is the index number at the beginning of the Initial Transference Intervention (ITI)?________

3. ITI is the first therapist-patient interaction in the transcript. YES NO

4. ITI is the last therapist-patient interaction in the transcript YES NO

5. What is the category of the ITI? _________

**Timing –of the Initial Transference Intervention (ITI)**

6. To what degree does the therapist’s ITI connect naturally to the preceding clinical material, such as content and time

context, allusions to the transference and other relevant issues? 0 1 2 3 4

7. How precise and striking is the therapist’s ITI? 0 1 2 3 4

**Category of the Transference Interventions (TI) in the Transference Work (TW)a**

8. Does the TW include TI of category 1? YES NO

9. Does the TW include TI of category 2? YES NO

10. Does the TW include TI of category 3? YES NO

11. Does the TW include TI of category 4? YES NO

12. Does the TW include TI of category 5? YES NO

*If more than one Transference Intervention (TI) in the Transference Work (TW), please score questions 13 and 14. If not, continue with question 15:*

**Timing –of the first Transference Intervention (TI) with the highest category score in the Transference Work (TW)**

13. To what degree does the therapist’s TI connect naturally to the preceding clinical material, such as content and time

context, allusions to the transference and other relevant issues? 0 1 2 3 4

14. How precise and striking is the therapist’s TI? 0 1 2 3 4

**Content in the Transference Work (TW)**

15. To what degree does the therapist refer to the patient’s relation to others? 0 1 2 3 4

16. To what degree does the patient refer to the patient’s relation to others? 0 1 2 3 4

17. To what degree does the therapist refer

to the patient’s relation to parental figures? 0 1 2 3 4

18. To what degree does the patient refer to the

patient’s relation to parental figures? 0 1 2 3 4

19. To what degree does the therapist point out

the patient’s attempt to avoid themes in the session

in order to control unpleasant emotions and thoughts? 0 1 2 3 4

20. To what degree does the therapist refer to the patient’s symptoms? 0 1 2 3 4

21. To what degree does the patient refer to the patient’s symptoms? 0 1 2 3 4

**Valence – in the Transference Work (TW)**

22. To what degree does the therapist make use of supportive interventions? 0 1 2 3 4

23. To what degree is the therapist challenging in the interventions? 0 1 2 3 4

**Response – in the Transference Work (TW)**

24. To what degree does the patient

express associations and/or self reflections in the TW? 0 1 2 3 4

25. To what degree does the patient show active cooperative engagement? 0 1 2 3 4

26. Identify with the patient:

What is the highest level of emotional involvement? 0 1 2 3 4
